# Review: Lessons Learned From Clinical Trials Using Antimicrobial Peptides (AMPs)

**DOI:** 10.3389/fmicb.2021.616979

**Published:** 2021-02-22

**Authors:** Gabrielle S. Dijksteel, Magda M. W. Ulrich, Esther Middelkoop, Bouke K. H. L. Boekema

**Affiliations:** ^1^Association of Dutch Burn Centres, Beverwijk, Netherlands; ^2^Department of Plastic, Reconstructive and Hand Surgery, Amsterdam Movement Sciences, Amsterdam UMC, Vrije Universiteit Amsterdam, Amsterdam, Netherlands; ^3^Department of Pathology, Amsterdam UMC, Vrije Universiteit Amsterdam, Amsterdam, Netherlands

**Keywords:** antimicrobial peptide, clinical trial, resistance, infection, cytotoxicity, mechanism of action, improvement strategies, peptide modifications

## Abstract

Antimicrobial peptides (AMPs) or host defense peptides protect the host against various pathogens such as yeast, fungi, viruses and bacteria. AMPs also display immunomodulatory properties ranging from the modulation of inflammatory responses to the promotion of wound healing. More interestingly, AMPs cause cell disruption through non-specific interactions with the membrane surface of pathogens. This is most likely responsible for the low or limited emergence of bacterial resistance against many AMPs. Despite the increasing number of antibiotic-resistant bacteria and the potency of novel AMPs to combat such pathogens, only a few AMPs are in clinical use. Therefore, the current review describes (i) the potential of AMPs as alternatives to antibiotics, (ii) the challenges toward clinical implementation of AMPs and (iii) strategies to improve the success rate of AMPs in clinical trials, emphasizing the lessons we could learn from these trials.

## Introduction

Antibiotic resistance is a global concern in health care as (new) resistance mechanisms are emerging and spreading globally. Resistant bacterial strains have been identified for various antibiotics in clinical use. For example, shortly after the emergence of penicillin-resistant *Staphylococcus aureus* in 1940 (Abraham and Chain, 1940), several pathogenic bacteria became resistant not only to penicillin but also to semi-synthetic penicillin, cephalosporins and newer carbapenems (Kumarasamy et al., 2010). In addition, the decline in the approval of new antibiotics by regulatory bodies has further exacerbated this problem.

As alternatives to antibiotics, AMPs have been at the forefront of international efforts because they are less likely to induce bacterial resistance (Wimley and Hristova, 2011). AMPs are a diverse group of naturally occurring peptides of the innate defense system with activity against various pathogens such as yeast, fungi, viruses and bacteria (Zasloff, 2002; Beisswenger and Bals, 2005). To inactivate these pathogens, AMPs display a multi-hit, non-specific and rapid action, resulting in the slow or limited emergence of resistance (Wimley and Hristova, 2011). Additionally, some AMPs show synergistic interactions with conventional antibiotics (Steenbergen et al., 2009; Wu et al., 2020), which could decrease the selection of antibiotic resistant bacteria.

Around the same time as the discovery of the first antibiotic penicillin in 1928, the first AMP nisin was discovered in milk (Zhang and Zhong, 2015). This AMP was approved by the FDA of the United States as a food preservative in 1988 due to its heat stability and tolerance of low pH (Delves-Broughton et al., 1996; Cleveland et al., 2001). After the discovery of nisin, several other AMPs such as gramicidin, tyrocidine, alamethicin, purothionin, and defensins were isolated from bacteria, fungi, plants, invertebrates and vertebrates (Van Epps, 2006; Bahar and Ren, 2013). However, the clinical application of AMPs as antimicrobials was limited due to toxicity considerations and other problems, such as high production costs as compared to antibiotics (Fry, 2018).

The renewed interest in AMPs as a consequence of the increasing number of antibiotic resistant and tolerant bacteria has resulted in the FDA approval of gramicidin and polymyxin B as constituents in Neosporin^®^ in 1955, colistin (polymyxin E) in 1962 and daptomycin in 2003 (Chen and Lu, 2020). Several naturally occurring and synthetic AMPs have been clinically investigated to combat pathogenic bacteria but since the approval of daptomycin no new AMPs have been approved as antimicrobials. To understand this innovation gap, we reviewed the literature to describe the potential of AMPs as alternative to antibiotics and the challenges toward clinical application of AMPs. Additionally, we provide an overview of the strategies that are currently available to facilitate the successful clinical implementation of AMPs using examples from clinical trials.

## Function of AMPs

### Physiological Role of AMPs in the Skin

**FIGURE 1:**
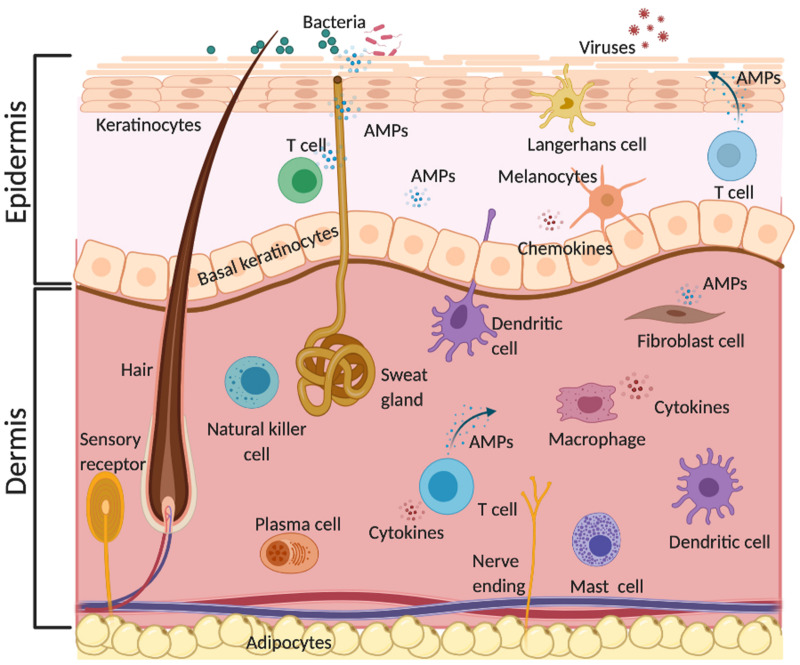
Physiological role of AMPs in the skin. AMPs are produced by different resident skin cells. They act as the first line of innate immune defense against various pathogens such as bacteria, fungi, and viruses via direct and indirect antimicrobial activities and/or immunomodulatory effects. This illustration was created with BioRender.com.

The skin is not only a physical barrier to the external environment. It is an active immune organ protecting the host from harmful toxins and pathogenic organisms (Salmon et al., 1994). The immune response of the skin involves various resident cells in the epidermis such as keratinocytes, melanocytes, Langerhans cells and γδ T cells, and in the dermis such as dendritic cells, macrophages, fibroblasts, mast cells, B and T cells, plasma cells and natural killer cells (Zasloff, 2002; Ryu et al., 2014; Lacey et al., 2016). These skin cells release several pro-inflammatory cytokines such as IL-17 and IL-22 and produce AMPs, which act as the first line of defense against microorganisms (Figure 1) (Liang et al., 2006). AMPs display a broad-spectrum of antimicrobial activity against yeast, fungi, viruses and bacteria. For example, the human cathelicidin LL-37 shows activity against various Gram-positive and Gram-negative bacteria, and antibiotic-resistant bacterial strains (Dean et al., 2011; Shurko et al., 2018). The same AMP also shows activity against fungi and some viruses such as influenza and HIV. Bergman et al. (2007) showed that LL-37 inhibits HIV-1 replication and suggested that this AMP contributes to the protection against HIV-1 infection. Furthermore, Luo et al. (2019) reported that LL-37 inhibits *Aspergillus fumigatus* infection via direct antifungal activity and reduction of excessive inflammation.

The ability to modulate the immune responses has been reported for several AMPs. LL-37 is a well-studied AMP with such immunomodulatory properties in humans. It acts as a chemoattractant for monocytes and promotes the production and release of various cytokines and chemokines that may direct the course and intensity of inflammation (Agier et al., 2015). Among others, LL-37 can reduce the inflammatory response via interaction with TLR. TLRs are widely expressed receptors on immune cells that recognize pathogenic-associated molecular patterns. LL-37 downregulates signaling through TLR4 by scavenging its ligand LPS (Larrick et al., 1995; Rosenfeld et al., 2006) as well as by disrupting the receptor complex function (Di Nardo et al., 2007; Brown et al., 2011). Furthermore, LL-37 potentially elongates the lifespan of neutrophils via the suppression of neutrophil apoptosis (Nagaoka et al., 2012), thereby enhancing host immunity. Additionally, Carretero et al. (2008) report that LL-37 activates and promotes angiogenesis and migration of keratinocytes, which results in an improved re-epithelialization and granulation tissue formation.

The antimicrobial and immunomodulatory effects of AMPs are necessary to maintain homeostasis of the skin function. Therefore, the production of AMPs is upregulated upon injury and infection (Miller et al., 2005; Sørensen et al., 2006). For example, in acne vulgaris several AMPs such as LL-37 and hBD-2 are upregulated by, e.g., keratinocytes in response to the *Propionibacterium acnes* (Nagy et al., 2006). As the *P. acnes* strains vary in their ability to stimulate inflammatory responses, upregulation of AMPs could be beneficial due to their antimicrobial and anti-inflammatory effects. In some skin conditions, for example in diabetic foot ulcers, the upregulation of AMPs such as hBD-2, 3, and 4 is often not sufficient to control the inflammation and wound infection (Rivas-Santiago et al., 2012). Therefore, such skin conditions require specialized care for proper healing.

### Structure and Mechanism of Action

Antimicrobial peptides are usually small, consisting of 12–50 amino acids. They are composed of hydrophilic, hydrophobic and cationic residues (net charge +2 to +11). The cationicity and hydrophobicity of AMPs are critical for bactericidal activity. Together with the hydrophilic residues, the hydrophobic residues form an amphipathic structure for insertion into the bacterial membrane (Ebenhan et al., 2014). To form this structure, some AMPs (i.e., α-helical peptides such as melittin) undergo conformational changes upon interaction with bacterial membranes, while others already have a rigid amphipathic structure (i.e., β-sheet peptides such as β-defensins) to target bacterial membranes (Ebenhan et al., 2014). The positive charge of AMPs facilitates the initial binding of AMPs to the membrane surfaces via electrostatic interactions. Bacterial membranes consisting of negatively charged phospholipid headgroups such as phosphatidylglycerol, cardiolipin, or phosphatidylserine show high affinity for cationic AMPs (Matsuzaki, 1999). Contrarily, mammalian cell membranes that are enriched with zwitterionic phospholipids such as phosphatidylethanolamine, phosphatidylcholine, or sphingomyelin show low affinity for cationic AMPs due to their neutral net charge.

**FIGURE 2:**
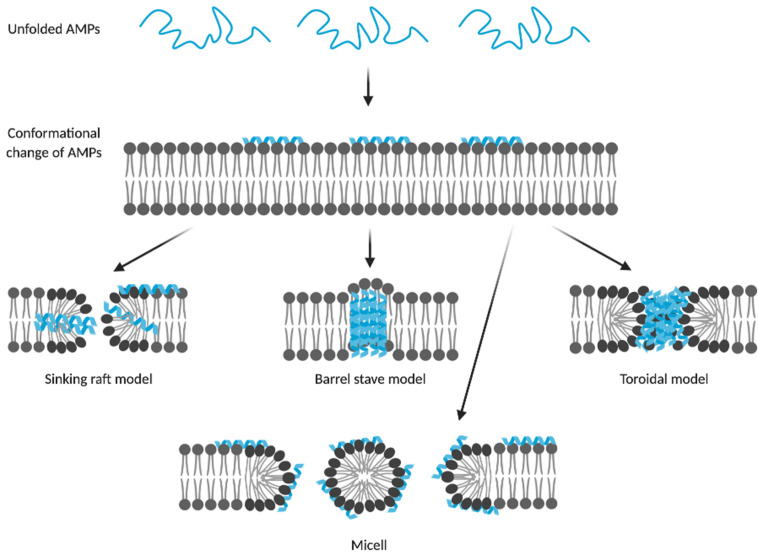
Membrane disruptive models of AMPs. Membrane disruptive AMPs form micelles or pores on the bacterial membrane, resulting in leakage of the intercellular components and cell death. Membrane pores can be categorized by their membrane topology and geometry into the sinking raft, barrel stave and toroidal models. AMPs that form barrel stave and toroidal pores display a transmembrane topology, whereas AMPs of the sinking raft model cause self-aggregation to sink into the membrane, creating transient pores. AMPs that form toroidal pores are mainly driven by electrostatic interactions and as a result of these interactions, the pores are covered by phosphate headgroups. AMPs forming micelles show a detergent-like mechanism of action, causing membrane perturbation. This illustration was created with BioRender.com and Bahar and Ren, 2013.

Most AMPs cause membrane disruption through non-specific interactions with the membrane surface. They are suggested to form micelles or pores, causing loss of membrane integrity and consequently leakage of intracellular components, resulting in cell death (Chan et al., 2006). Depending on the membrane topology and geometry of pores, pores can be described by three models, i.e., barrel-stave, toroidal and sinking-raft model (Figure 2). In the sinking-raft model, AMPs lie on the membrane surface and cause an increase in membrane curvature. Self-aggregation of the peptide causes the AMPs to sink into the membrane, creating transient pores (Brogden, 2005). In both the barrel-stave pore and toroidal pore, the peptide has a transmembrane topology. The main difference between these two models is that the formation of barrel-stave pores is driven by both hydrophobic and electrostatic interactions, whereas the formation of toroidal pores is mainly driven by electrostatic interactions (Bertelsen et al., 2012). As a result, toroidal pores are covered by phosphate headgroups, initiating changes in membrane curvature. The mechanism of action of AMPs that form micelles is not well-understood. It is believed that these AMPs act by a detergent-like mechanisms causing intrinsic perturbations of the membrane (Li et al., 2017).

Membrane disruptive AMPs might also kill bacteria using non-membrane disruptive pathways, and vice versa (Hale and Hancock, 2007). Additionally, they could act independently or in synergy with non-membrane disruptive AMPs. Non-membrane disruptive AMPs are able to transverse membranes to reach their intracellular target components. Such AMPs could inhibit protein-folding, proteases, cell division, the synthesis and metabolism of proteins, nucleic acids and cell walls (Le et al., 2017). Previously, Edgerton et al. (2000) showed that two AMPs with clearly different structures, i.e., histatin 5 and human neutrophil (HNP)-1, act on similar pathways. The role of these AMPs suggests that membrane and non-membrane disruptive AMPs serve as equally important peptides of the innate defense system to inactivate pathogens.

## Clinical Trials Using AMPs

The results from pre-clinical studies using AMPs revealed that AMPs could be used for the prevention and treatment of various clinical conditions. For example, peptide coating of catheters using a novel peptide E6 prevented catheter associated infections in mouse models (Yu et al., 2017). PXL150 demonstrated efficacy against *Pseudomonas aeruginosa* in burn wounds in mouse models (Björn et al., 2015). Both LL-37 and IDR-1 restored pulmonary function in mice with pneumonia (Hou et al., 2013). Also, nisin demonstrated *in vitro* efficacy against *Clostridium difficile* (Bartoloni et al., 2004). Furthermore, several AMPs including LL-37 displayed anti-biofilm activity *in vivo* (Chennupati et al., 2009; Segev-Zarko et al., 2015; Batoni et al., 2016). Based on promising pre-clinical results, numerous AMPs have been investigated in human clinical trials to demonstrate efficacy and safety. Several of these trials are still ongoing, others are completed, discontinued or approved. The successes and flaws of these clinically investigated AMPs are described in the following sections. Structural information, mechanism of action and the intended target of AMPs in clinical trials is provided in Table 1.

**TABLE 1:** Clinical trial(s) of AMPs under investigation and in clinical use.

Peptide name	Description	Target	Administration	Phase	Clinical Trial ID	Mechanism	MW (g/mol)	Length	Net charge	References
**Nisin**	Polycyclic lantibiotic	Gram-positive bacteria	Oral		NCT02928042; NCT02467972	Depolarization of cell membrane	3354	34	4	Prince et al., 2016
**Gramicidin**	Polycyclic peptide	Infected wounds and ulcers	Topical	III	NCT00534391	Membrane disruption/immunomodulation	1882	16	0	David and Rajasekaran, 2015
**Polymyxin B**	Cyclic polypeptide	Gram-negative bacteria	Topical	III	NCTOO490477; NCT00534391	Membrane disruption/immunomodulation	1204	10	5	Morrison and Jacobs, 1976
**Polymyxin E (Colistin)**	Cyclic polypeptide	*A. baumannii/*pneumonia	Intravenous	III	NCT01292031; NCT02573064	Membrane disruption/immunomodulation	1155	10	5	Yu et al., 2015
**Daptomycin**	Lipopeptide	Skin infection/bacteremia	Intravenous	III	NCT01922011; NCT00093067; NCT01104662; NCT02972983	Membrane disruption/immunomodulation	1621	13	0	Taylor and Palmer, 2016
**LL-37**	Human cathelicidin	Leg ulcers	Topical	II	EUCTR2012-002100-41	Membrane disruption/immunomodulation	4491	37	6	Brown et al., 2011
**Melittin**	α-helical peptide	Inflammation	Intradermal	I/II	NCT02364349, NCT01526031	Membrane disruption/immunomodulation	2846.5	26	5	Lee and Bae, 2016
**Friulimicin**	Cyclic lipopeptide	MRSA/pneumonia	Intravenous	I	NCT00492271	Membrane disruption	1303.5	12	−2	Schneider et al., 2009
**Murepavadin (POL7080)**	Analog of Protegrin	*P. aeruginosa, K. pneumoniae*	Intravenous	II	EUCTR2017-003933-27-EE	Binding to LptD	1553.8	14	5	Srinivas et al., 2010
**Neuprex^®^ (rBPI21)**	Derivative of BPI	Pediatric meningococcemia	Intravenous	III	NCT00462904	Membrane disruption	∼21000	193		Schultz and Weiss, 2007
**Iseganan (IB-367)**	Analog of Protegrin	Pneumonia/oral mucositis	Topical	III	NCT00118781; NCT00022373	Membrane disruption	1900.3	17	4	Orlov et al., 1805
**Surotomycin (CB-315)**	Cyclic lipopeptide	*C. difficile*	Oral	III	NCT01597505	Membrane disruption	1680.8	13	−3	Alam et al., 2015
**Pexiganan (MSI-78)**	Analog of Magainin	Diabetic foot ulcers	Topical	III	NCT00563394; NCT00563433; NCT01590758; NCT01594762	Membrane disruption/immunomodulation	2477.2	22	9	Gottler and Ramamoorthy, 2009
**XOMA-629 (XMP-629)**	Derivative of BPI	Impetigo/acne rosacea	Topical	III		Immunomodulation	1158.4	9	3	Easton et al., 2009
**Omiganan (MBI-226)**	Derivative of Indolicidin	Antisepsis/catheter infection	Topical	III	NCT00231153; NCT00608959	Membrane disruption/immunomodulation	1779.2	12	5	Rubinchik et al., 2009
**NVB-302**	Lantibiotic	*C. difficile*	Oral	I	ISRCTN40071144	Inhibition of cell wall synthesis	1754.0	19	0	Crowther et al., 2013
**OP-145**	Derivative of LL-37	Chronic middle ear infection	Ear drops	I/II	ISRCTN84220089	Membrane disruption/immunomodulation	3093.8	24	6	Malanovic et al., 2015
**P113 (PAC-113)**	Fragment of Histatin-5	Oral candidiasis	Mouth rinse	II	NCT00659971	Membrane disruption/immunomodulation	1564.8	12	5	Woong et al., 2008
**LTX-109**	Synthetic tripeptide	MRSA/impetigo	Topical	I/II	NCT01803035; NCT01158235	Membrane disruption	788.1	3	2	Isaksson et al., 2011; Sivertsen et al., 2014
**EA-230**	Oligopeptide	Sepsis	Intravenous	II	NCT03145220	Immunomodulation	415.5	4	0	Van Groenendael et al., 2018
**SGX942 (Dusquetide)**	Analog of IDR-1	Oral mucositis	Oral rinse	III	NCT03237325	Immunomodulation	553.7	5	2	Kudrimoti et al., 2016
**hLF1-11**	Fragment of human lactoferrin	Bacterial/fungal infections	Intravenous	I/II	NCT00430469	Membrane disruption/immunomodulation	1373.7	11	4	Nibbering et al., 2001; Welling et al., 2018
**C16G2**	Synthetic peptide	*Streptococcus mutans*	Mouth wash	II	NCT03004365	Membrane disruption	4077.4	35	10	Guo et al., 2015
**Novexatin (NP213)**	Cyclic Cationic peptide	Fungal nail infection	Topical	II	NCT02933879	Membrane disruption	1093.3	7	7	Mercer et al., 2020
**Ramoplanin (NTI-851)**	Glycolipodepsipeptide	*C. difficile*, VRE	Oral	III		Inhibition of cell wall synthesis	2568.1	17	2	Fulco and Wenzel, 2006
**p2TA (AB103)**	Synthetic peptide	Necrotic tissue infection	Intravenous	III		Immunomodulation	1037.2	8	−1	Bulger et al., 2014
**D2A21**	Synthetic peptide	Burn wound infections	Topical	III		Membrane disruption	2775.4	23	9	Muchintala et al., 2020
**Melamine**	Chimeric peptide	Contact lenses microbials	Topical	II/III		Membrane disruption	2786.6	29	15	Yasir et al., 2019, 2020
**Mel4**	Derivative of melamine	Contact lenses microbials	Topical	II/III	ACTRN1261500072556	Membrane disruption	2347.8	17	14	Yasir et al., 2020
**LFF571**	Semisynthetic thiopeptide	*C. difficile*	Oral	II	NCT01232595	Inhibition of protein synthesis	1366.6			Leeds et al., 2012
**Delmitide (RDP58)**	Derivative of HLA	Inflammatory bowel disease	Topical	II	ISRCTN84220089	Immunomodulation	1228.6	10	2	Travis et al., 2005
**DPK-060**	Derivative of Kininogen	Acute external otitis	Ear drops	II	NCT01447017	Membrane disruption/immunomodulation	2503.2	20	7	Håkansson et al., 2019
**GSK1322322 (Lanopepden)**	Synthetic hydrazide	Bacterial skin infection	Oral	II	NCT01209078	Peptide deformylase inhibitor	479.3			Peyrusson et al., 2015
**PXL01**	Analog of Lactoferrin	Postsurgical adhesions	Topical	II	NCT01022242	Immunomodulation	3061.6	25	5	Edsfeldt et al., 2017
**AP-214**	Derivative of α-MSH	Post-surgical organ failure	Intravenous	II	NCT00903604	Membrane disruption/immunomodulation	2433.9	19	7	Doi et al., 2008
**PMX-30063 (Brilacidin)**	Defensin mimetic	Acute bacterial skin infection	Intravenous	II	NCT01211470; NCT02052388	Membrane disruption/immunomodulation	936.9			Mensa et al., 2014
**XF-73 (Exeporfinium chloride)**	Derivative of porphyrin	*Staphylococcal* infection	Topical	II	NCT03915470	Membrane disruption	765.8			Ooi et al., 2009
**CZEN-002**	Derivative of α-MSH	Antifungal	Topical	II		Immunomodulation	971.2	8	2	Csato et al., 1989
**Ghrelin**	Endogenous peptide	Chronic respiratory infection	Intravenous	II	NCT00763477	Immunomodulation	3314.9	28	5	Gualillo et al., 2003
**Wap-8294A2 (Lotilibcin)**	Produced by *Lysobacter species*	Gram-positive bacteria	Topical	I/II		Membrane disruption	1562.8	12	1	Itoh et al., 2017
**PL-5**	Synthetic peptide	Skin infections	Topical	I		Membrane disruption	2933.5	26	6	Miyake et al., 2004
**IDR-1**	Bactenecin	Infection prevention	Intravenous	I		Immunomodulation	1391.7	13	3	Yu et al., 2009

*A. baumannii; Acinetobacter baumannii; BPI, bactericidal permeability increasing protein; C. difficile, Clostridium difficile; HLA, human leukocyte antigen; IDR, innate defense regulator; K. pneumoniae, Klebsiella pneumoniae; LptD, lipopolysaccharide transport protein D; MRSA, methicillin-resistant Staphylococcus aureus; MSH, melanocyte-stimulating hormone; P. aeruginosa, Pseudomonas aeruginosa; VRE, vancomycin-resistant Enterococci.*

### AMPs Approved for Clinical Use

**FIGURE 3:**
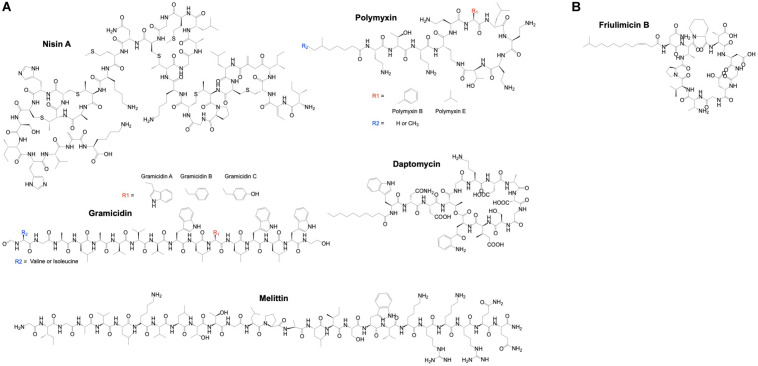
Chemical structure of AMPs in clinical use. **(A)** A few α-helical (gramicidin and melittin) and cyclic AMPs (nisin A, polymyxin and daptomycin) have been approved for clinical use. **(B)** Despite the physiochemical similarities of the cyclic AMP friulimicin B with daptomycin, the Phase I clinical trial of friulimicin B was terminated due to unfavorable pharmacokinetics. ChemDraw version 19 was used to draw the chemical structure of AMPs.

Currently, nisin, gramicidin, polymyxins, daptomycin and melittin are in clinical use as alternative to antibiotics because of their antimicrobial potency (Figure 3A). Nisin, also known as nisin A, is composed of 34 amino acids and consists of dehydrated, unsaturated and thioether amino acids, forming five lanthionine rings. It is naturally produced by lactic acid bacteria such as *Lactococcus lactis* and shows a broad-spectrum of bactericidal activity (Stevens et al., 1991; Severina et al., 1998). *L. lactis* also produces nisin Z, F and Q, which differ by up to 10 amino acids from nisin A resulting in differences in physiochemical properties and antimicrobial activity (Piper et al., 2011). Among others, nisins inhibit cell wall synthesis through interactions with lipid II, a precursor molecule that is essential for bacterial cell-wall bio-synthesis. Nisins also form membrane pores causing cell lysis (Prince et al., 2016). Nisin A is approved as a food preservative and is GRAS (Delves-Broughton et al., 1996; Cleveland et al., 2001). In clinical trials, the effect of nisin A has been investigated using probiotics, i.e., the consumption of live microorganisms (e.g., *L. lactis*) that produce nisin A. The results of a systematic review of such trials indicated that these probiotics reduce infectious complications and may subsequently reduce intensive care unit mortality (Petrof et al., 2012). Minor but possible side effects of nisin A are itching (pruritus) and flushing of the skin, and nausea or vomiting. The safety profile together with the broad-spectrum of bactericidal activity, indicated that the application of nisin could extend beyond food-related bacteria (Blay et al., 2007; Shin et al., 2015). Applications of nisins in humans include dental-care and pharmaceutical products such as for the treatment of stomach ulcer and colon infections (Sakamoto et al., 2001; Mitra et al., 2019).

Gramicidin or gramicidin D is a mixture of gramicidin A, B and C making up 80, 6, and 14% of the mixture, respectively. These AMPs are hydrophobic linear polypeptides composed of 15 amino acids (Meikle et al., 2016). They are naturally produced by Gram-positive *Brevibacillus brevis* commonly found in soil (Van Epps, 2006). Gramicidins form ion-channels within the bacterial membrane, allowing the passive diffusion of Na^+^ and K^+^ along their concentration gradient (David and Rajasekaran, 2015). This results in membrane depolarization, osmotic swelling and lysis of bacterial cells. Gramicidin is effective against a variety of Gram-positive bacteria and is clinically used for ophthalmic purposes as a constituent in Neosporin^®^. In a clinical trial, patients suffering from hordeolum who received the ophthalmic solution containing gramicidin (Neosporin^®^) reported a comparable pain score as those who received a placebo treatment (Hirunwiwatkul and Wachirasereechai, 2005). Additionally, the duration of cure of these treatment groups was not statistically different (*p* = 0.988). The authors of this study suggested that the lack of statistically significant differences between the placebo and peptide-treated group could be due to the small sample size of 14 patients in each group (Hirunwiwatkul and Wachirasereechai, 2005). In another clinical study using a larger sample size of 91 patients, the effect of this ophthalmic solution on the duration of cure of bacterial-positive corneal ulcers was reported (Bosscha et al., 2004). An average of 12.5 days was required for complete re-epithelialization of these ulcers. This was more favorable compared to the duration of cure of ulcers treated with ofloxacin (13.7 days) and ciprofloxacin (14.4 days) (Prajna et al., 2001). These findings suggest that Neosporin^®^ containing gramicidin could be used as an alternative to conventional antibiotics for such ophthalmic purposes. Another AMP in Neosporin^®^ is polymyxin B.

Polymyxins (A, B, C, D, and E) are a group of cyclic polypeptides naturally produced by Gram-positive *Paenibacillus polymyxa*. They show activity against MDR Gram-negative bacteria such as *P. aeruginosa* and *Escherichia coli* (Zavascki et al., 2007). Polymyxins bind to the lipid A component of LPS on the outer membrane of Gram-negative bacteria (Morrison and Jacobs, 1976), which contributes to the insertion of the AMPs into the membrane. They can increase cell-permeability via a detergent-like mechanism, which causes cell death (Schroder et al., 1992). Polymyxins in clinical use are polymyxin B and E, which differ only by one amino acid from each other (Li et al., 2005; Falagas et al., 2006; Kwa et al., 2007). Polymyxin B is prescribed to treat eye infections, whereas polymyxin E is used to treat wound infections. These AMPs are recognized as crucial but last-resort treatment options because of their ability to induce adverse events. Nephrotoxicity and neurotoxicity are the most common adverse events reported for polymyxins (Falagas and Kasiakou, 2006; Cisneros et al., 2019). To optimize the clinical use of polymyxins without such severe adverse effects, clinical trials are currently being executed, e.g., in combination with different antimicrobial agents. Minor side effects, such as blurred vision, watery eyes, and sensitivity to light, have been reported for Neosporin^®^ containing two AMPs, gramicidin and polymyxin B, and an aminoglycoside antibiotic neomycin. Thus, not one agent but the mixture of three antimicrobial agents are responsible for these minor side effects.

Daptomycin is a cyclic lipopeptide consisting of 13 amino acids, which is naturally produced by the bacterium *Streptomyces roseosporus* (Ball et al., 2004). It shows bactericidal activity against Gram-positive bacteria, including antibiotic resistant strains (Jorgensen et al., 2003). Daptomycin inhibits cell wall synthesis, causes membrane depolarization and forms membrane pores, eventually causing cell death. A daily treatment of 4-6 mg/kg daptomycin is recommended in critically ill patients. For the treatment of bacteria with reduced susceptibility high dosages of 10 mg/kg can be prescribed, which are also well tolerated. In a phase I clinical trial, 2 of 5 healthy volunteers who received 4 mg/kg per 12 h of daptomycin (8 mg/kg per day) developed reversible myopathy (Dvorchik et al., 2003). Nonetheless, the number of incidence and the severity of myopathy were substantially decreased in healthy volunteers when the total dose of 8 mg/kg was administered once daily. Other studies reported that the development of myopathy was not related to the administration of daptomycin but to other factors such as concomitant medications, comorbidities and the number of surgical interventions (Galar et al., 2019). In a Phase IV clinical trial, the effect of daptomycin on the resolution of skin infections was compared to that of the standard of care, i.e., cloxacillin, nafcillin, oxacillin, flucloxacillin or vancomycin (Arbeit et al., 2004). The success rate of the daptomycin-treated patients was 71.5%, which was clinically and statistically comparable to the standard treatments with a success rate of 71.1%. In another clinical study, efficacy of daptomycin was demonstrated in a placebo-control trial. All patients also received a β-lactam therapy. In this study, the daptomycin treatment resulted in faster clearance of bacteremia than the control treatment (placebo + β-lactam therapy) (Cheng et al., 2018). Hence, combination therapy using daptomycin can improve the clinical success rate.

Melittin is the predominant (40–48%) component of venom from the European honeybee *Apis mellifera*. It is composed of 26 amino acids and adopts an α-helical conformation upon interaction with the membrane surface (Terwilligert and Eisenbergg, 1982). It possesses anti-inflammatory properties (Lee and Bae, 2016) and is therefore approved by the FDA for relieving pain and swelling associated with rheumatoid arthritis, tendinitis, bursitis and multiple sclerosis (Son et al., 2007; Alves et al., 2011). Melittin also forms membrane toroidal pores to inactivate pathogens. This was shown in several *in vitro* and animal experiments using cancer cells (Gajski and Garaj-Vrhovac, 2013), viruses (Memariani et al., 2020) and (resistant) bacteria (Park et al., 2006; Van Den Bogaart et al., 2008; Choi et al., 2015). Hence, similar to nisin, the clinical application of melittin could extend beyond the FDA-approved purposes. Side effects of melittin are redness and swelling of the skin at the side of administration, itching, trouble breathing, nausea, sleepiness and low blood pressure.

### Flaws of Some AMPs

Some clinical trials using AMPs were discontinued or terminated due to various reasons. For example, the Phase I clinical trial of friulimicin B in healthy volunteers was terminated due to its unfavorable pharmacokinetic profile. The nature of these unfavorable findings remains unknown and unexpected. Friulimicins (A, B, C, D, and E) are a group of naturally occurring peptides produced by *Actinoplanes friuliensis* and like daptomycin, they are cyclic lipopeptides (Figure 3B). Friulimicin B was clinically investigated and demonstrated efficacy in various murine infection models (Endermann et al., 2007). It has similar physiochemical properties and mechanism of action as daptomycin (Schneider et al., 2009) but has been found to trigger different stress responses in *Bacillus subtilis* as compared to daptomycin (Wecke et al., 2009). This might be related to differences in the pharmacokinetic profiles of these AMPs. Another AMP, Murepavadin (POL7080) failed unexpectedly in advanced clinical trials. Murepavadin is a 14 amino acid cyclic peptide that targets the LPS transport protein D (LptD) on the bacterial membrane to form pores (Srinivas et al., 2010). It was demonstrated to be safe in Phase I clinical trials in healthy volunteers and in subjects with an impaired renal function (Martin-Loeches et al., 2018). Safety and efficacy of murepavadin were demonstrated in Phase II clinical trials in patients with acute exacerbation of non-cystic fibrosis bronchiectasis or ventilator-associated bacterial pneumonia due to *P. aeruginosa* (Martin-Loeches et al., 2018). However, Phase III clinical trial of this AMP in patients with nosocomial pneumonia was prematurely ended due to higher than expected acute kidney injuries, i.e., 56% for the murepavadin plus ertapenem treated group versus 25–40% for the meropenem treated control group and according to the literature (BioSpace, 2019).

Besides pharmacokinetic and safety issues, several AMPs have failed Phase III clinical trials because of lack of clear efficacy or lack of superiority over conventional treatments. A clear example is demonstrated for the AMP Neuprex^®^ (rBPI21) which is a recombinant α-helical peptide consisting of the first 193 amino acids of the N-terminus of BPI. Clinical studies showed that patients with meningococcemia or hemorrhage due to trauma who received Neuprex^®^ had no toxic side effects and showed a trend toward improved outcomes, i.e., reduced bone marrow aplasia and deaths (Demetriades et al., 1999; Guinan et al., 2011). However, Neuprex^®^ failed to show clear efficacy (*p* = 0.07) as compared to the placebo-treated group (Giroir et al., 2001). Similar to Neuprex^®^, at least five AMP that have completed advanced clinical trials, failed to show clear efficacy (i.e., iseganan and XOMA-629) or superiority over conventional treatments (i.e., surotomycin, pexiganan, and omiganan) (Gordon et al., 2005). Nevertheless, the latter AMPs could be potential alternatives to conventional antibiotics due to their favorable safety profile and low or limited ability to induce bacterial resistance. Since antibiotics are no longer routinely used to treat bacterial infections as a consequence of resistance development, the ability of AMPs to induce bacterial resistance, is a more important parameter to consider during clinical trials. Hence, instead of superiority trials, equivalence or non-inferiority trials in which AMPs cause a similar effect as the standard treatment, should become more common (Committee for Proprietary Medicinal Products, 2001). In two equivalence trials with systemic ofloxacin as the comparator, efficacy of topical pexiganan was determined in patients with diabetic foot ulcers (Lipsky et al., 2008). The combined results of these trials demonstrated that pexiganan was clinically comparable to this antibiotic. However, equivalence to ofloxacin was not acceptable as main evidence of efficacy and FDA approval of pexiganan. Additional clinical trials were required to demonstrate efficacy superior to a topical placebo cream plus standard treatment for diabetic foot ulcers. Of note, clinical trials are often not designed using placebo treatment only as control due to ethical reasons. In the additional trials, pexiganan plus standard treatment failed to meet the primary outcome, i.e., resolution of infection (Genetic Engineering, and Biotechnology News, 2016). Failure of AMPs in such trials may arise from stability issues, inappropriate drug administration or unknown interactions between the peptide and the standard treatment. Currently, the developers of pexiganan continue to evaluate the data to consider this peptide for the treatment of other clinical indications.

### Challenges Toward Clinical Application of AMPs

The development of AMPs for clinical use is accompanied by several challenges such as high development and production costs, cytotoxic issues, reduced activity in clinically relevant environments and the emergence of bacterial resistance, despite the initial claims that they may not induce resistance. To begin with, the manufacturing costs of antibiotics are relatively inexpensive. For example, aminoglycoside production costs $0.80 per gram as compared to $50–400 per gram of amino acid for AMPs by solid phase synthesis (Marr et al., 2006). As a consequence, alternative methods are required to promote commercial-scale production.

Furthermore, AMPs acting on membranes are not completely selective to microbial cells and may be toxic for eukaryotic cells as well. Several AMPs cause hemolytic and/or cytotoxic effects at antimicrobial concentrations, limiting their wider utilization (Laverty, 2014; Bacalum and Radu, 2015). Polymyxins are an example of such AMPs: they are crucial antimicrobials to eradicate MDR Gram-negative bacteria but they may cause nephrotoxicity and neurotoxicity at antimicrobial concentrations (Falagas and Kasiakou, 2006).

Another drawback for the clinical implementation of AMPs is the low antimicrobial activity in clinically relevant environments. AMPs may lose their bactericidal activity under physiological salt conditions due to loss of electrostatic interactions between AMPs and cell membranes (Falanga et al., 2016; Mohamed et al., 2016). In the presence of serum, AMPs may bind to proteins such as albumin (Sivertsen et al., 2014; Li et al., 2017). Additionally, AMPs can be susceptible to proteolytic degradation (Perona and Craik, 1997; Thwaite et al., 2006; McCrudden et al., 2014). Also, Starr et al. (2016) suggested that host cells can interfere with the activity of AMPs in a way similar to serum protein binding. This reduces the effective concentration of available AMPs to eradicate bacteria.

Although AMPs do not seem to induce bacterial resistance, resistance to AMPs has been reported. AMPs that require specific recognition molecules such as LPS, Lipid A, Lipid I/II and LptD, on the membrane surface of bacteria most likely develop resistance. For example, resistance to nisin involves mutations in bacterial cells that induce changes in membrane and cell wall composition and eventually prevents the binding of nisin to lipid II (Kramer et al., 2008). Alternatively, bacteria may inactivate nisin using dehydropeptide reductase, also known as nisinase (Bastos et al., 2015). Resistance to polymyxins and cross-resistance to AMPs have also been reported (Li and Nation, 2006; Valencia et al., 2009; Arcilla et al., 2016; Dobias et al., 2017). Resistance to polymyxins is mediated by the *mrc-1* gene encoding a phosphoethanolamine modification in lipid A, which prevents the initial binding of polymyxins to the bacterial membranes (Liu et al., 2016). This gene was initially isolated from Chinese livestock animals and has been identified in the human fecal microbiome, indicating that polymyxin resistance is horizontally transferable (Arcilla et al., 2016). Moreover, Li et al. (2007) reported that the *aps* AMP sensor/regulator system is important for *S. aureus* virulence *in vivo.* They show that AMPs may induce resistance mechanisms in MRSA via this system, which involves the D-alanylation of teichoic acids, the incorporation of lysophosphatidylglycerol in the bacterial membrane, the increase of lysine biosynthesis and AMP transport systems (Arcilla et al., 2016). Although the impact of bacterial resistance on the minimal inhibitory concentration of the AMPs (2–30-fold increase) is less dramatic than for antibiotics (100–1000-fold increase) (Andersson et al., 2016), the risk of bacterial resistance should be carefully investigated.

## Improvement Strategies

**FIGURE 4:**
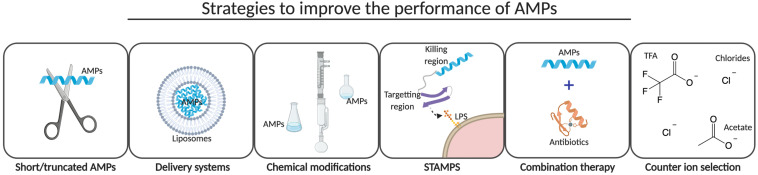
Improvement strategies. Several strategies have been developed to reduce production costs and cytotoxic effects and to improve bio-availability and efficacy of AMPs. Short and/or truncated AMPs have been pursued by several companies to reduce the production costs, whereas the development of a delivery system and the introduction of chemical modifications are more common to improve the bio-availability and efficacy of AMPs *in vivo*. STAMPS, combination therapy with conventional antibiotics and the selection of a counter ion during the final step of peptide synthesis might not only improve efficacy but may also reduce cytotoxic effects of AMPs. This illustration was created with BioRender.com.

The majority of the AMPs under clinical evaluation are positively charged analogs of naturally occurring AMPs and are limited to topical or intravenous applications for an effective bio-available concentration of the peptides. Importantly, the route of drug administration could markedly affect the efficacy of AMPs as efficacy is dependent on the bio-distribution and stability of the peptides (Benet, 1978). Analogs of naturally occurring AMPs have been prepared to overcome the challenges associated with high production costs, low bio-availability and efficacy, and cytotoxic effects of AMPs (Figure 4). Strategies to improve the performance of AMPs are described in the following sections.

### Ultra-Short and/or Truncated AMPs

Efforts to reduce production costs include alternative peptide synthesis methods and the production of ultra-short and/or truncated AMPs. The latter has been pursued by several companies. The AMPs OP-145 (Nell et al., 2006), P113 (Woong et al., 2008), LTX-109 (Midura-Nowaczek and Markowska, 2014), and EA-230 (Van Groenendael et al., 2018) all consist of a lower number of amino acids as compared to their “original” peptide LL-37, histatin-5, bovine lactoferrin and loop-2 of β-hCG, respectively. Beside truncation of AMPs, the synthesis of ultra-short AMPs such as the 5-amino acid linear peptide SGX942 further reduces the production costs of AMPs (Kudrimoti et al., 2016). Alternatively, solution phase synthesis or by chemoenzymatic methods could be used for production of small AMPs (Bray, 2003). This remains challenging for large peptides and therefore a biotechnological approach is often considered, i.e., the production of AMPs in microorganisms (Ingham and Moore, 2007). Magainin, hBD-3, melittin and other AMPs have been synthesized using calmodulin as carrier protein (Ishida et al., 2016; Boto et al., 2018). This protein protects the producing bacterial cells, e.g., *E. coli* from the toxic effects of the AMPs and prevents degradation of the AMP during the production process. Alternative approaches to obtain AMPs from plants or bacterial ribosomes have been reviewed by Montesinos and Bardají (2008), and Rogers and Suga (2015), respectively.

### Delivery Systems

To improve the bio-availability of AMPs, delivery systems can be used to administer the peptides. Nisin is readily degraded by enzymes in the gastrointestinal tract (Heinemann and Williams, 1966). To target *C. difficile*, a bacterium that can infect the colon (Le Lay et al., 2016), nisin requires a delivery vehicle to reach the colon without being digested and absorbed by the upper gastrointestinal tract. To achieve this, nisin has been encapsulated in pectin/HPMC compression coated tablets to form an enzymatically controlled delivery system (Ugurlu et al., 2007). Alternative systems such as liposomes, nanoparticles, and nisin-controlled gene expression in *Lactobacillus gasseri* have also shown to be successful delivery systems (Neu and Henrich, 2003; Taylor et al., 2007; Khan and Oh, 2016). Colon-specific delivery approaches such as pro-drugs and conjugates have been reviewed by Fang et al. (2017) and Mishra et al. (2017). These approaches have also been used to improve the *in vivo* bio-availability of different AMPs, for example Polymyxin E, which is administered as an inactive pro-drug that undergoes hydrolysis to release the active AMPs. Polymyxin E was also successfully integrated into hydrogels for the treatment of burn wound infections (Zhu et al., 2017). Please note that such delivery systems, may not only improve the bio-availability of the AMPs but may also improve the efficacy and reduce cytotoxicity, as a consequence of increased solubility and specificity, respectively (Mahlapuu et al., 2016; Kim et al., 2017; Nordström and Malmsten, 2017).

### Chemical Modifications

The order and position of amino acids were found to play an important role in the biological activity of the hLT-1-1 peptide, according to its structure-activity relationship (Welling et al., 2018). Also, the α-helical content, the hydrophobicity and amphipathicity of AMPs may affect their bactericidal activity and cytotoxicity. Schmidtchen et al. (2014) reported that an increase in the hydrophobicity might induce hemolytic activity of AMPs. Another group showed that a reduction in the net positive charge of AMPs may not affect the bactericidal efficacy of AMPs but may reduce cytotoxic effects (Jiang et al., 2008). For higher bactericidal activity and less cytotoxic effects, the introduction of arginine was found to be superior to lysine for providing the positive charge of AMPs (Yang et al., 2018). To improve proteolytic stability, different approaches have been used such as the introduction of D-amino acids, cyclization, amidation or acetylation of the terminal regions (Strömstedt et al., 2009; Gentilucci et al., 2010; Chu et al., 2013). To increase the salt and serum stability of AMPs, tryptophan or β-naphthylalanine end-tagging of the terminal regions of AMPs could be considered (Chu et al., 2013; Pfalzgraff et al., 2018).

### Specifically Targeted AMPs (STAMPS)

Due to the broad-spectrum activity and non-specific mechanisms of action of AMPs, these peptides may induce cytotoxic effects as well. To reduce these effects, STAMPS have been designed (Eckert et al., 2012). STAMPS selectively target and kill a specific pathogenic species without affecting the normal flora (Eckert et al., 2006). They consist of at least two regions, i.e., one or multiple targeting regions and a killing region linked by a spacer. The targeting region improves the activity of the AMPs by enhancing the initial binding of the peptide to the specific pathogenic determinants on the membrane (He et al., 2010). Using two are more targeting regions reduces the likelihood of bacterial resistance and improves efficacy (Sarma et al., 2018). Currently, C16G2 which is a synthetic AMP or STAMP, is under clinical investigation for the treatment of tooth decay by *Streptococcus mutans*. The N-terminus of C16G2, is the targeting region for *S. mutans* and the C-terminus is the killing region or AMP G2 (Kaplan et al., 2011). C16G2 demonstrated a strong safety profile and efficacy against *S. mutans* (Todd and Pierre, 2015).

### Combination Therapy

To improve treatment outcomes, two or more antibiotics are often used in clinical practice. The same could be done for novel AMPs as many peptides show synergistic interactions with conventional antibiotics. This could not only reduce the amount of peptide needed for effective treatment and thus reduce costs but may also extend the lifetime of current antibiotics (Phee et al., 2015; Kampshoff et al., 2019). There are numerous examples of AMPs demonstrating synergism (Rand and Houck, 2004; Oo et al., 2010; Dosler et al., 2016; Alni et al., 2020). The combination of polymyxins with carbapenems or rifampicin suppresses the development of polymyxin resistance (Rodriguez et al., 2010; Lenhard et al., 2016). Also, Polymyxin B is used in combination with gramicidin and neomycin in Neosporin^®^ due to their synergistic interactions, resulting in reduced resistance development and less cytotoxic effects (Booth et al., 1994; Tempera et al., 2009).

### Counter-Ion Selection

The final step of AMP synthesis, which involves the cleavage and deprotection of the peptide chain with, e.g., TFA should be investigated to improve efficacy and reduce cytotoxicity. Counter-ions such as TFA anions are able to interact with positively charged AMPs and affect the hydrogen-bonding network along with the secondary structure (Blondelle et al., 1995; Gaussier et al., 2002). Also, TFA was shown to be cytotoxic for mammalian cells (Cornish et al., 1999). Previously, Sikora et al. (2018) studied the effect of three counter-ions, i.e., TFA anions, acetate and chlorides, on the bactericidal efficacy and cytotoxicity of a set of AMPs. They found that the peptide salts of acetate and chlorides seemed to be more potent antimicrobials than trifluoroacetates. However, trifluoroacetates have greater ability to promote α-helix formation in, e.g., LL-37 (Johansson et al., 1998). Additionally, acetate counter-ions seemed to be associated with high hemolytic activity (Sikora et al., 2018). In contrast, pexiganan acetate showed less cytotoxicity in cell viability assays and was the most stable salt for pexiganan (Desai, 2013; Sikora et al., 2018). Hence, superiority of one salt over another is peptide-dependent and should be taken into account.

## Conclusion and Perspectives

As a result of the increasing number of antibiotic resistant bacteria, there has been a renewed interest in AMPs as a potential alternative to conventional antibiotics. AMPs display clear advantages over conventional antibiotics to combat various infectious diseases. In particular, (i) their broad spectrum of activity, (ii) multi-hit, non-specific and rapid mode of action, which results in limited emergence of resistance, (iii) the potential immunomodulatory properties and (iv) synergistic interactions with conventional antibiotics could eliminate the threat of MDR bacteria. Yet, until now, only a few AMPs (e.g., nisin, gramicidin, polymyxins, daptomycin, and melittin) have reached the clinic. Challenges toward clinical application of AMPs include cytotoxic effects, production costs, and problems related to peptide bio-availability and efficacy. To overcome these challenges, several strategies have been designed such as the preparation of ultra-short/truncated AMPs, delivery systems and STAMPS, chemical modifications and the careful selection of a counter-ion in the final step of AMP synthesis. Although not all AMPs in the clinical pipeline will reach the market, these strategies could improve the success rate of AMPs in clinical trials. Nonetheless, several AMPs in clinical trials have failed due to lack of clear efficacy or superiority over conventional antibiotics, while showing a trend toward improved clinical outcomes. Therefore, practical strategies should also be considered in future clinical testing of AMPs as we have learned the following lessons:

(1) The application of AMPs can extend beyond FDA-approved clinical indications;
(2) Defining the most optimal dose and administration regimen might reduce cytotoxic effects of AMPs;
(3) Efficacy of AMPs can be demonstrated in equivalence or non-inferiority trials with an antibiotic as comparator;
(4) Bacterial resistance development should be included as one of the primary outcome parameters in clinical trials of AMPs;
(5) The bio-availability and efficacy of AMPs can be improved using delivery systems and,
(6) The combination AMPs with conventional antibiotics or other compounds (e.g., AMPs) might result in an improved antimicrobial effect in clinical trials.

Taking these lessons into consideration, an increasing number of AMPs could reach the market as multi-functional, potent and long-lasting antimicrobials against various infectious diseases.

## Author Contributions

GD wrote a draft version of the manuscript. MU, EM, and BB contributed to the design of the review and revised the manuscript. All authors assisted with the interpretation of the findings, read, and approved the final manuscript.

## Conflict of Interest

The authors declare that the research was conducted in the absence of any commercial or financial relationships that could be construed as a potential conflict of interest.

## Abbreviations

AMPs: antimicrobial peptides
BPI: bactericidal permeability-increasing protein
β-hCG: human β-chorionic gonadotropin
FDA: Food and Drug Administration
GRAS: generally recognized as safe
hBD: human β-defensin
hLT-1-1: human lactoferrin
HNP: human neutrophil
HPMC: hydroxypropyl methylcellulose
IL: interleukin
LPS: lipopolysaccharide
LptD: lipopolysaccharide transport protein D
MDR: multi-drug resistant
STAMPS: specifically targeted antimicrobial peptides
TFA: trifluoroacetic acid
TLR: toll-like receptors.
